# Development of Bi- and Tri-Layer Nanofibrous Membranes Based on the Sulfated Polysaccharide Carrageenan for Periodontal Tissue Regeneration

**DOI:** 10.3390/md21110565

**Published:** 2023-10-28

**Authors:** Stefanos Kikionis, Konstantina Iliou, Aikaterini G. Karra, Georgios Polychronis, Ioannis Choinopoulos, Hermis Iatrou, George Eliades, Efthymia Kitraki, Ioulia Tseti, Spiros Zinelis, Efstathia Ioannou, Vassilios Roussis

**Affiliations:** 1 Section of Pharmacognosy and Chemistry of Natural Products, Department of Pharmacy, National and Kapodistrian University of Athens, Panepistimiopolis Zografou, 15771 Athens, Greece; skikionis@pharm.uoa.gr (S.K.); ilioukonstantina@outlook.com (K.I.); eioannou@pharm.uoa.gr (E.I.); 2 Department of Basic Sciences, School of Dentistry, National and Kapodistrian University of Athens, 11527 Athens, Greece; aikaterini.g.karra@gmail.com (A.G.K.); ekitraki@dent.uoa.gr (E.K.); 3 Department of Biomaterials, School of Dentistry, National and Kapodistrian University of Athens, 11527 Athens, Greece; gpolisg@yahoo.gr (G.P.); geliad@dent.uoa.gr (G.E.); szinelis@dent.uoa.gr (S.Z.); 4 Industrial Chemistry Laboratory, Department of Chemistry, National and Kapodistrian University of Athens, Panepistimiopolis Zografou, 15771 Athens, Greece; ichoinop@chem.uoa.gr (I.C.); iatrou@chem.uoa.gr (H.I.); 5 Uni-Pharma S.A., 35 Kalyftaki Str., 14564 Kifissia, Greece; jtsetis@uni-pharma.gr

**Keywords:** GTR/GBR membranes, periodontitis, periodontal tissue regeneration, carrageenans, marine sulfated polysaccharides, nanofibers

## Abstract

Periodontitis is a microbially-induced inflammation of the periodontium that is characterized by the destruction of the periodontal ligament (PDL) and alveolar bone and constitutes the principal cause of teeth loss in adults. Periodontal tissue regeneration can be achieved through guided tissue/bone regeneration (GTR/GBR) membranes that act as a physical barrier preventing epithelial infiltration and providing adequate time and space for PDL cells and osteoblasts to proliferate into the affected area. Electrospun nanofibrous scaffolds, simulating the natural architecture of the extracellular matrix (ECM), have attracted increasing attention in periodontal tissue engineering. Carrageenans are ideal candidates for the development of novel nanofibrous GTR/GBR membranes, since previous studies have highlighted the potential of carrageenans for bone regeneration by promoting the attachment and proliferation of osteoblasts. Herein, we report the development of bi- and tri-layer nanofibrous GTR/GBR membranes based on carrageenans and other biocompatible polymers for the regeneration of periodontal tissue. The fabricated membranes were morphologically characterized, and their thermal and mechanical properties were determined. Their periodontal tissue regeneration potential was investigated through the evaluation of cell attachment, biocompatibility, and osteogenic differentiation of human PDL cells seeded on the prepared membranes.

## 1. Introduction

Periodontitis is a microbially induced inflammation of the periodontium that is characterized by the destruction of the periodontal ligament (PDL) and alveolar bone [1,2]. It constitutes the principal cause of teeth loss in adults, affecting 20–50% of the population worldwide [3]. Due to its high prevalence, the reduction of oral health-related quality of life [4] and the huge healthcare economic cost [5], it is considered as a major public health issue that needs to be addressed vigorously [6].

The optimal target of reestablishing tooth functionality is achieved with periodontal regeneration procedures that try to restore the damaged periodontal apparatus to its initial state [2,3]. Periodontal tissue regeneration treatment has shown remarkable results in the reconstruction of periodontal tissue and is based on the utilization of guided tissue/bone regeneration (GTR/GBR) membranes that act as a physical barrier, isolating the fast-growing epithelium and providing adequate time and space for PDL cells and osteoblasts to proliferate and counter effect attachment loss [1,3,7,8]. For their desired optimum performance, GTR/GBR membranes must have specific essential properties. These include biocompatibility, cell-occlusiveness, tissue integration effects, space-maintaining capability, and clinical manageability [9,10]. A major drawback of the currently available GTR/GBR membranes is the low predictability of attachment and bone gains [11].

Recently, nanofibers have emerged as a new approach in periodontal tissue engineering [2,3], as nanofibrous scaffolds designed to resemble the natural architecture of the extracellular matrix (ECM) have been shown to enhance cell proliferation and differentiation and are nowadays developed as carriers and support structures for cells, growth factors, bioactive, and antiseptic/antimicrobial substances [12,13,14]. Electrospinning is a versatile technique and the most successful method used for the preparation of ultrafine fibers with sizes ranging from the micrometer to the nanometer scale, and it exhibits great potential for fabricating membranes for periodontal regeneration [5,15,16]. The major advantages of micro/nanofibrous membranes include the high porosity and increased surface area, the tunable fiber orientation and size, and the favorable micromechanical properties [17]. Furthermore, their intrinsic interconnectivity and topography can facilitate cell attachment, offering better spatial organization for cell growth and enabling the exchange of waste and nutrients [16,18]. Tailor-made electrospun nanofibrous matrices combining the mechanical strength of synthetic polymers with the high bioactivity of natural polymers can efficiently enhance cellular function, allowing for the development of novel GTR/GBR membranes with significant tissue regeneration properties [16,19].

Among natural polymers, marine biopolymers are highly biocompatible and biodegradable materials, featuring diverse structures and functionalities [20,21]. The wide spectrum of bioactivities exhibited by marine sulfated polysaccharides renders them ideal biomaterials for the development of novel systems for tissue engineering, wound healing, and drug delivery applications [22,23,24,25,26]. Possessing tunable and multifunctional characteristics, marine polysaccharides-based electrospun nanofibrous scaffolds have attracted recently increasing attention for the development of advanced systems for periodontal tissue engineering [27]. Carrageenans are anionic sulfated polysaccharides present in the cell walls of red seaweeds [23]. They are composed of alternating units of D-galactose and 3,6-anhydro-D-galactose linked by α-(1,3) and β-(1,4) glycosidic bonds. Based on the 3,6-anhydro-D-galactose content and the sulfation degree, they are classified into three main types, namely kappa (κ)-carrageenans, iota (ι)-carrageenans, and lambda (λ)-carrageenans [24]. Possessing antioxidant, antiviral, anticoagulant, anti-hyperlipidemic, immunomodulatory, and antitumor activities, they have been widely exploited in the cosmetics, food, and pharmaceutical industries [28]. Due to their significant biological properties, low cost, high biocompatibility and biodegradability, and chemical analogy to glycosaminoglycans of the native ECM, carrageenans have attracted significant interest for their utilization in tissue engineering applications, with κ- and ι-carrageenans showing significant osteogenic potential [29,30,31]. Formulated in electrospun nanofibers, they are ideal candidates for the regeneration of periodontal tissue, since they have shown great potential for bone regeneration by promoting the attachment and proliferation of osteoblasts [32,33].

Poly(L-glutamic acid), formed by amide bond linkage of the naturally occurring L-glutamic acid, is a non-toxic synthetic polypeptide devoid of immunogenicity or antigenicity [34]. Due to its biodegradability and high hydrophilicity, it has been used in tissue engineering applications showing significant chondrogenic tissue regeneration potential when combined with natural biopolymers of marine origin, such as alginates and chitosan [35,36,37]. Furthermore, poly(L-glutamic acid) is a promising biomaterial for oral care applications considering that L-glutamic acid residues are responsible for the enamel demineralization inhibitory effect of statherin, a calcium-binding salivary protein that acts as a source of free calcium ions in the oral cavity, promoting the remineralization of tooth enamel [38].

Among synthetic polymers, polycaprolactone (PCL) is a biodegradable and biocompatible synthetic polyester, broadly used in biomedical applications [39,40]. Exhibiting low immunogenicity, good mechanical strength, and high elasticity, it is often used in periodontal tissue regeneration and treatment of oral diseases, as it can maintain a stable oral environment without producing acidic degradation byproducts [15,41]. However, its high hydrophobicity hinders cell response as it lacks surface cell adhesion and proliferation characteristics required by GTR membranes [42,43]. Blends with natural polymers with high osteo-inductive ability and surface modification can improve its cellular behavior, enhancing its periodontal regeneration potential [44,45,46,47,48,49].

Considering the complex anatomy of the periodontium, comprising PDL and alveolar bone, multiphasic/multilayer GTR membranes have been developed in recent years, aiming to regulate the responses of both soft and hard tissues during the healing process [11,50,51]. Consisting of different natural or/and synthetic biodegradable and biocompatible polymers in each phase/layer displaying specific functionalities, they can synergistically enhance cellular activity, promote tissue regeneration, and potentially reconstruct the entire periodontium, restoring its healthy state [52,53,54].

In the framework of our research investigations towards the development of marine biopolymer-based scaffolds for wound healing and tissue engineering applications [49,55,56,57,58,59,60], we have focused on the development of carrageenan-based GTR nanofibrous membranes for the treatment of periodontitis and bone loss. Bi- and tri-layer nanofibrous GTR membranes based on carrageenan and other biocompatible polymers were designed, fabricated, and evaluated for their efficacy in periodontal tissue regeneration. In all cases, the fabricated GTR membranes consisted of an outer layer of a cast PCL film. In the case of the tri-layer membranes, the middle layer was composed of electrospun nanofiber blends of PCL and calcium salt of poly(L-glutamic acid) (PG-Ca), while the inner layer comprised electrospun nanofibers based on calcium or sodium salt of carrageenan (CG-Ca or CG-Na, respectively). As far as the bi-layer membranes are concerned, the inner layer consisted of electrospun nanofiber blends of PCL and CG-Ca or PG-Ca, or a combination of them. The fabricated membranes were morphologically characterized, and their thermal and mechanical properties were determined. Their periodontal regeneration potential was investigated through the evaluation of cell attachment, biocompatibility, and osteogenic differentiation of human PDL cells seeded on the prepared membranes.

## 2. Results and Discussion

### 2.1. Design of the GTR Nanofibrous Membranes

In the present study, in order to evaluate the potential of carrageenans for periodontal tissue regeneration, we designed and fabricated nanofibrous GTR membranes based on carrageenans in combination with PG-Ca and other biocompatible polymers. The fabricated membranes were designed to act (a) as a barrier preventing the epithelial cells from infiltrating and proliferating and (b) as a source of calcium ions promoting the osteoblasts’ growth. It was envisaged that carrageenans, which have been proven to promote attachment and proliferation of osteoblasts [29,30,31,32,33], would enhance bone and PDL regeneration, whereas the release of calcium ions from the fibers would promote mineralization and bone growth.

The developed membranes consisted of two or three layers. In both cases, the membranes consisted of a cast outer layer, providing mechanical strength to the membrane, blocking at the same time the epithelial cells from reaching the PDL area. For the outer layer, PCL, which is a biocompatible hydrophobic polymer, was chosen since it degrades slower than the other polymers used, offering ample time for bone and PDL regeneration. The tri-layer membranes included a middle layer to support bone regeneration and an inner layer to promote bone and PDL regeneration, while the bi-layer membranes consisted of an inner layer to support bone regeneration, as well as to promote PDL regeneration.

Initially, in order to investigate the efficacy of carrageenans in periodontal tissue regeneration, the tri-layer GTR1 and GTR2 membranes were fabricated and evaluated. GTR1 and GTR2 membranes were composed of electrospun nanofibers of PG-Ca in the middle layer and electrospun nanofibers based on CG-Ca or CG-Na, respectively, in the inner layer. In a further step, motivated by the better osteo-inductive performance of the GTR1 membrane, the efficacy of CG-Ca was investigated in a simpler assembly through the preparation and evaluation of the bi-layer GTR3 membrane, comprising both CG-Ca and PG-Ca in its inner layer. Moreover, in order to compare the contribution of CG-Ca to that of PG-Ca to the osteo-inductive ability of the fabricated membranes, the bi-layer GTR4 and GTR5 membranes, comprising in the inner layer electrospun nanofibers of only CG-Ca or only PG-Ca, respectively, were prepared and evaluated (Table 1).

For the electrospinning of CG-Ca, CG-Na, PG-Ca, and CG-Ca/PG-Ca, polyethylene oxide (PEO) was added as an easily spinnable and non-toxic polymeric carrier in order to enhance their electrospinnability into nonwoven membranes, contributing also to the hydrophilicity of the membranes with its hydrophilic character. For the preparation of the middle layer of the tri-layer membranes and the inner layer of the bi-layer membranes, the corresponding polymers were co-electrospun with PCL in order to fabricate a composite fibrous network that would ensure the necessary cohesion with the cast outer layer, enhancing the stability of the nanofibrous network in aqueous environments.

### 2.2. Characterization of the Fabricated GTR Membranes

**Figure 1:**
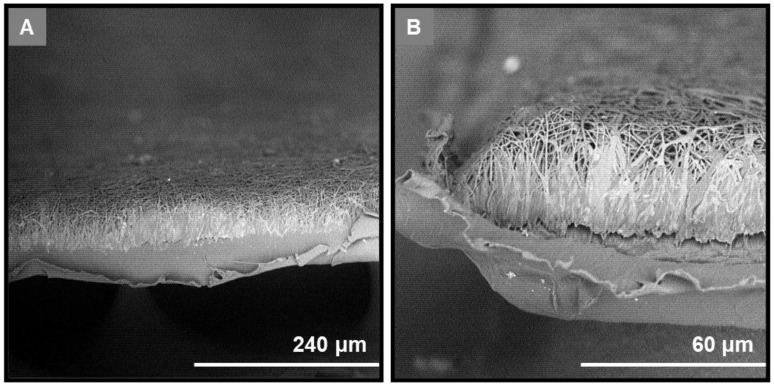
SEM images of the electrospun fibers on the surface of the cast outer layer of the bi-layer GTR3 membrane. (**A**) Image showing the side of the membrane at 500x magnification. (**B**) Image showing the edge of the membrane at 2000× magnification.

**Figure 2:**
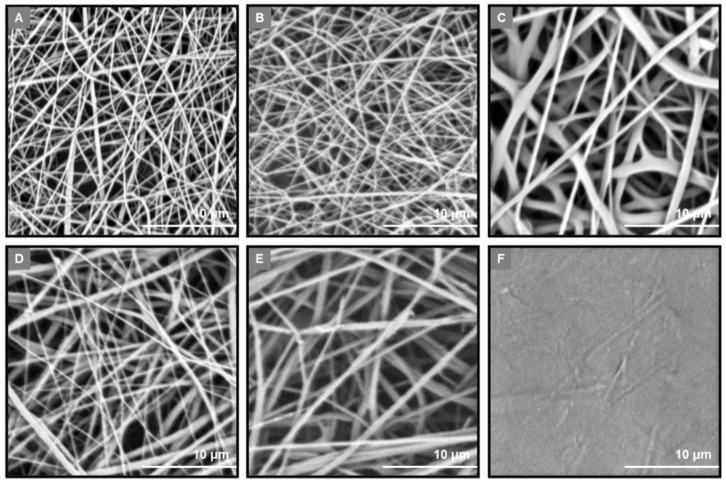
SEM images of the (**A**) inner layer of the GTR1 membrane, (**B**) inner layer of the GTR2 membrane, (**C**) inner layer of the GTR3 membrane, (**D**) inner layer of the GTR4 membrane, (**E**) inner layer of the GTR5 membrane, and (**F**) cast outer layer (GTR0).

For the casting of the outer layer, the process was optimized to produce a smooth cast membrane on the surface of a rotating drum, whereas the electrospinning parameters were fine-tuned to obtain a uniform fibrous network with well-shaped fibers void of beads on the surface of the outer cast layer. The cast outer layer (GTR0) and the fibrous inner layers of the fabricated GTR membranes were morphologically evaluated through analyses of their SEM images. In all cases, the analysis of their morphology revealed the successful deposition of the electrospun fibers on the surface of the cast outer layer, exhibiting efficient cohesion between the outer and the electrospun fibrous layers. A non-porous surface was obtained in the case of the cast outer layer, which was covered by a dense fibrous network, resulting in a “grass-like” morphology (Figure 1). All GTR membranes exhibited a uniform fibrous web of randomly oriented fibers with the variation in fiber sizes attributed to the different polymer composition and/or electrospinning conditions (Figure 2).

**Figure 3:**
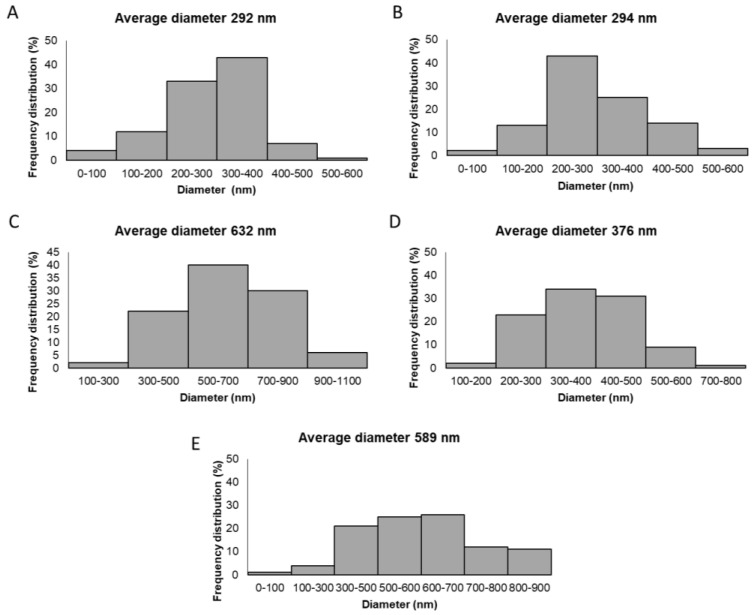
Diameter distribution histograms of (**A**) GTR1, (**B**) GTR2, (**C**) GTR3, (**D**) GTR4, and (**E**) GTR5 membranes.

In the case of the GTR1 membrane, the CG-Ca/PEO fibrous inner layer showed a uniform fibrous network consisting of smooth fibers of cylindrical morphology with size diameters ranging from 43 to 586 nm and an average diameter size of 292 ± 93 nm. The GTR2 membrane exhibited a similar fibrous web to that of the GTR1 membrane, revealing the fibrous layer of the CG-Na/PEO fibers with sizes ranging from 54 to 545 nm and an average diameter size of 294 ± 74 nm. In the case of the GTR3 membrane, the PCL and CG-Ca/PG-Ca/PEO fiber blends resulted in a uniform fibrous network with increased fiber sizes (ranging from 154 to 1180 nm with an average diameter of 632 ± 176 nm) in comparison to the rest of the GTR membranes, not only due to the presence of the PCL fibers, but also due to the different spinnability of CG-Ca and PG-Ca from the same spinning solution. The GTR4 membrane, composed of PCL fibers blended with CG-Ca/PEO fibers, exhibited cylindrical-shaped fibers of increased size (ranging from 131 to 705 nm and an average diameter size of 376 ± 112 nm) as compared to those of GTR1, due to the presence of the blended PCL fibers. The fibrous network of the GTR5 membrane, comprising blended PCL and PG-Ca/PEO fibers, exhibited smooth fibers of cylindrical shape with sizes ranging from 94 to 899 nm and an average diameter of 589 ± 137 nm. The diameter distribution histograms are shown in Figure 3.

**Figure 4:**
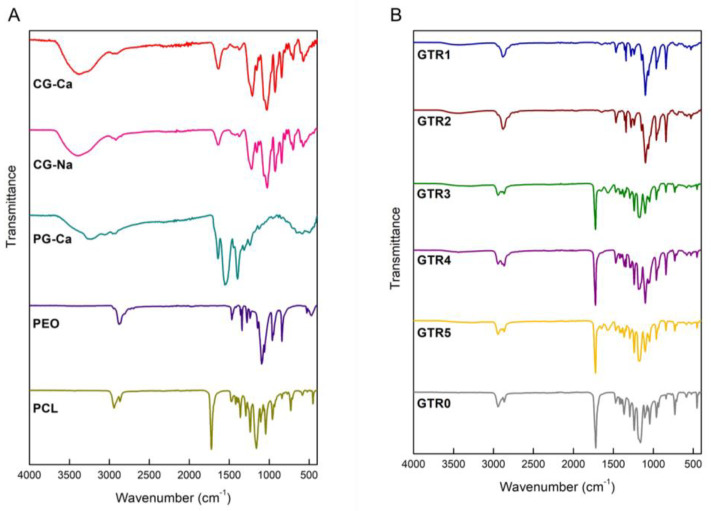
(**A**) FTIR spectra of the CG-Ca, CG-Na, PG-Ca, PEO, and PCL raw materials. (**B**) FTIR spectra of the GTR1, GTR2, GTR3, GTR4, GTR5, and GTR0 membranes.

The FTIR spectra of CG-Ca, CG-Na, PG-Ca, PEO, and PCL are shown in Figure 4A. The FTIR spectrum of CG-Ca displayed a broad absorption band at 3376 cm^−1^ which was assigned to the stretching vibrations of the -OH groups, a strong absorption band at 1213 cm^−1^ attributed to the -S=O stretching vibrations of the sulfate esters, as well as a very strong absorption band at 1030 cm^−1^ attributed to glycosidic linkage C-O-C stretching vibrations. The FTIR spectrum of CG-Na displayed a broad absorption band at 3392 cm^−1^ which was assigned to the stretching vibrations of the -OH groups, a strong absorption band at 1223 cm^−1^ attributed to the -S=O stretching vibrations of the sulfate esters, and a very strong absorption band at 1026 cm^−1^ attributed to glycosidic linkage C-O-C stretching vibrations. In the FTIR spectra of both CG-Ca and CG-Na, the strong absorption band at 926 cm^−1^ indicated the presence of 3,6-anhydro-D-galactose, while the absorption band at 845 cm^−1^ indicated the presence of D-galactose-4-sulphate. Finally, the weak absorption band at 805 cm^−1^ indicated the presence of 3,6-anhydro-D-galactose-2-sulphate.

In the FTIR spectrum of PG-Ca, the most characteristic absorption bands were observed at 3237 cm^−1^, attributed to the stretching vibrations of the overlapped signals of –OH and N–H groups, and at 1642 and 1553 cm^−1^, attributed to amide I (C=O stretching vibrations) and amide II (N-H bending vibrations) bands, respectively, indicating an α-helical conformation [61,62].

PEO exhibited the most characteristic absorption bands at 2878 cm^−1^, attributed to -CH_2_ stretching vibrations, and at 1094 cm^−1^, attributed to C-O-C stretching vibrations [63]. PCL exhibited asymmetric and symmetric stretching vibrations of -CH_2_ at 2943 and 2869 cm^−1^, a strong absorption band at 1726 cm^−1^ attributed to stretching vibrations of -C=O, and absorption bands at 1292 and 1160 cm^−1^ assigned to C-O and C-C stretching vibrations in crystalline and amorphous phases, respectively [64].

The FTIR spectra of the fabricated GTR membranes (Figure 4B) revealed the characteristic absorption bands of the ingredients comprising mainly their inner layer. The tri-layer GTR1 membrane exhibited a broad absorption band recorded at 3439 cm^−1^ that was assigned to the ‒OH stretching vibrations of CG-Ca, whereas the absorption band at 2883 cm^−1^ was attributed to the ‒CH_2_ stretching vibrations of PEO. The tri-layer GTR2 membrane exhibited a broad absorption band recorded also at 3439 cm^−1^ that was assigned to the ‒OH stretching vibrations of CG-Na, while the absorption band at 2880 cm^−1^ was attributed to the ‒CH_2_ stretching vibrations of PEO. In the FTIR spectrum of the bi-layer GTR3 membrane, a broad absorption band observed at 3335 cm^−1^ was attributed to the ‒OH stretching vibrations of CG-Ca, most probably overlapped with the –OH and N–H stretching vibrations of PG-Ca, whereas the absorption band of amide II of PG-Ca was evident at 1568 cm^−1^. The absorption band at 2895 cm^−1^ was attributed to the ‒CH_2_ stretching vibrations of PEO, whereas PCL was evident from its characteristic absorption bands of ‒CH_2_ asymmetric and symmetric stretching vibrations at 2943 and 2866 cm^−1^ and of ‒C=O stretching vibrations at 1725 cm^−1^. The FTIR spectrum of the bi-layer GTR4 membrane exhibited a broad absorption band at 3439 cm^−1^ attributed to the ‒OH stretching vibrations of CG-Ca, an absorption band at 2892 cm^−1^ attributed to the ‒CH_2_ stretching vibrations of PEO, and the characteristic absorption bands of PCL evident at 2946, 2868, and 1725 cm^−1^ attributed to the ‒CH_2_ asymmetric and symmetric stretching vibrations and the ‒C=O stretching vibrations, respectively. In the FTIR spectrum of the bi-layer GTR5 membrane, the presence of PG-Ca was evident from the overlapped signals of N–H and O–H at 3272 cm^−1^, and from the amide I and amide II absorption bands at 1647 cm^−1^ (C=O stretching vibrations) and 1570 cm^−1^ (N-H bending vibrations). The absorption band at 2895 cm^−1^ was attributed to the ‒CH_2_ stretching vibrations of PEO, whereas PCL was evident from the absorption bands of the ‒CH_2_ asymmetric and symmetric stretching vibrations at 2946 and 2866 cm^−1^ and of the ‒C=O stretching vibrations at 1725 cm^−1^. Finally, the FTIR spectrum of the cast membrane (GTR0) exhibited the characteristic signals of PCL.

**Figure 5:**
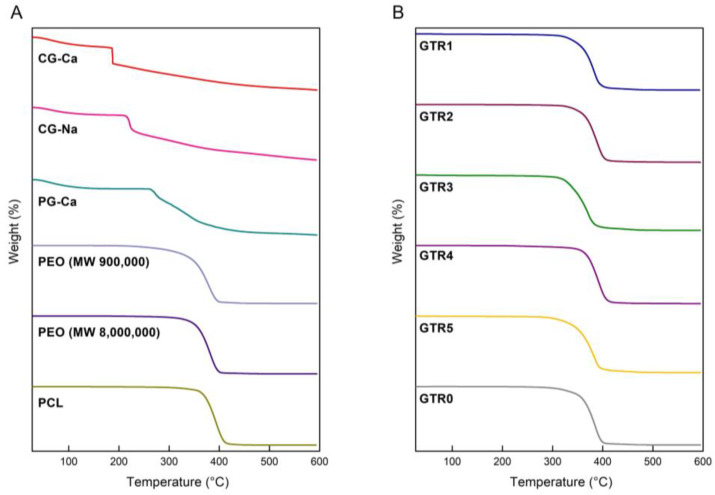
(**A**) TGA thermograms of the CG-Ca, CG-Na, PG-Ca, PEO (Mw 900,000), PEO (Mw 8,000,000), and PCL raw materials. (**B**) TGA thermograms of the GTR1, GTR2, GTR3, GTR4, GTR5, and GTR0 membranes.

The thermal stability of the fabricated membranes, as well as those of the utilized raw materials was investigated by TGA analysis. As observed in the TGA thermograms of the raw materials (Figure 5A), CG-Ca initially showed a slight mass loss up to 95 °C due to moisture volatilization, followed by a major decomposition step from 186 up to 188 °C. Similarly, CG-Na exhibited a main decomposition step from 215 to 225 °C after an initial moisture loss up to 105 °C. In the case of PG-Ca, moisture loss was observed up to 107 °C, followed by a main degradation step between 264 and 414 °C. PEO (Mw 900,000) and PEO (Mw 8,000,000) exhibited similar thermal profiles characterized by a main decomposition step occurring from 341 up to 398 °C and from 347 °C up to 400 °C, respectively, whereas the decomposition of PCL occurred from 365 to 411 °C. All GTR membranes exhibited similar thermal profiles due to the dominant presence of the PCL cast layer, characterized by a main degradation step in their thermogravimetric curves (Figure 5B), with the differences in their thermal degradation attributed to different synergistic degradation phenomena resulting from the different polymer composition and fibrous layer assembly. More specifically, the decomposition of the GTR1 membrane was initiated at 324 °C and completed at 400 °C, whereas the GTR2 membrane began to decompose at 348 °C and its degradation was completed at 406 °C. The decomposition of the GTR3 membrane was initiated at 315 °C and completed at 390 °C, while that of the GTR4 membrane started at 355 °C and was completed at 412 °C. Finally, the GTR5 membrane started to decompose at 310 °C and its degradation was completed at 394 °C, whereas the degradation of the GTR0 membrane occurred from 341 to 400 °C.

### 2.3. Determination of the Degradation Rate of the Fabricated GTR Membranes

All GTR membranes showed adequate stability up to 28 days of incubation at 37 °C in a simulated saliva solution, exhibiting limited weight loss rates (Figure S1). The cast membrane (GTR0) did not show any weight loss on day 28, indicating that the weight loss of the GTR membranes was due to the degradation of their fibrous layers. In the case of the GTR1 membrane, after a 13% weight loss on day 1, corresponding to a 56% weight loss of its fibrous layer, the weight loss reached approximately 17% on day 28, accounting for a 67% weight loss of its fibrous ingredients. The GTR2 membrane showed limited degradation during the study, with no significant weight changes from day 1 to day 28, averaging an 11% weight loss corresponding to a 58% weight loss of its fiber network. Similarly, no significant changes were observed from day 1 to day 28 in the weight of the GTR3 membrane, with the membrane exhibiting approximately an 11% weight loss corresponding to a 49% weight loss of fibers. The GTR4 membrane lost approximately 6% of its weight up to day 21 and this loss was slightly increased to 10% on day 28, corresponding to a 42% and 48%, respectively, weight loss of its fibrous layer. The GTR5 membrane exhibited an approximately 8% weight loss corresponding to 50% of its fibrous layer from day 1 to day 28, with no significant weight changes observed. In all cases, the fibrous layers remained attached on the surface of the cast outer layer during the degradation study, and no detachment phenomena were observed, indicating good cohesion between the inner/middle and the outer layers.

### 2.4. Release of Ca^+2^ from the Fabricated GTR Membranes

**Figure 6:**
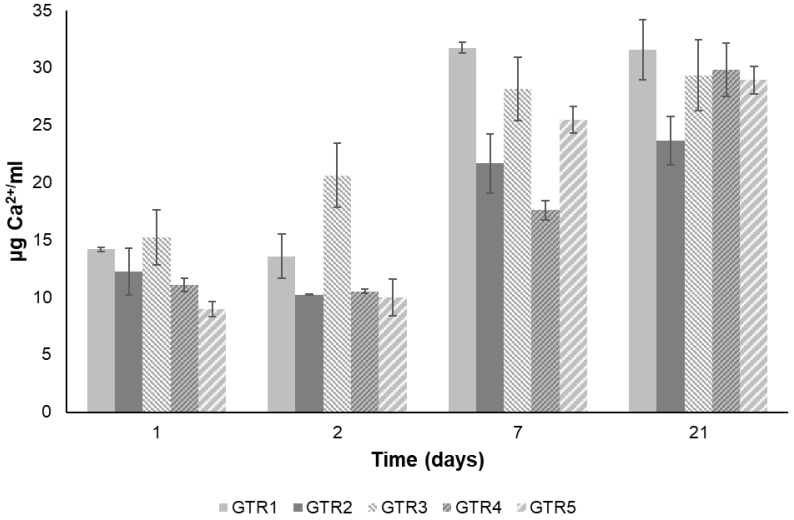
Release of Ca^+2^ from the GTR1, GTR2, GTR3, GTR4, and GTR5 membranes.

For the determination of Ca^+2^ release, the GTR membranes were immersed in an aqueous solution simulating saliva for different time intervals. The Ca^+2^ release rate from the fabricated membranes is shown in Figure 6. All GTR membranes exhibited a sustained release of Ca^+2^ for at least three weeks, demonstrating their potential to act as a continuous source of calcium ions for promoting osteoblast growth and bone regeneration.

More specifically, the concentration of Ca^+2^ released from the fabricated membranes in the saliva solution on day 1 ranged from 9.0 to 15.2 μg/mL, with the GTR5 membrane releasing the lowest amount. On day 2, the release of Ca^+2^ was at similar levels to those on day 1 and ranged from 10 to 20.6 μg/mL, with only the GTR3 membrane showing a measurable increase. On day 7 all membranes showed an increase, with the concentration of Ca^+2^ in the saliva solution ranging from 17.6 to 31.7 µg/mL and the GTR1 membrane releasing the highest amount. Finally, on day 21, the amount of released Ca^+2^ from the membranes ranged between 23.6 and 31.6 μg/mL, with only the GTR4 membrane showing a significant increase.

### 2.5. Determination of the Mechanical Properties of the Fabricated GTR Membranes

**Figure 7:**
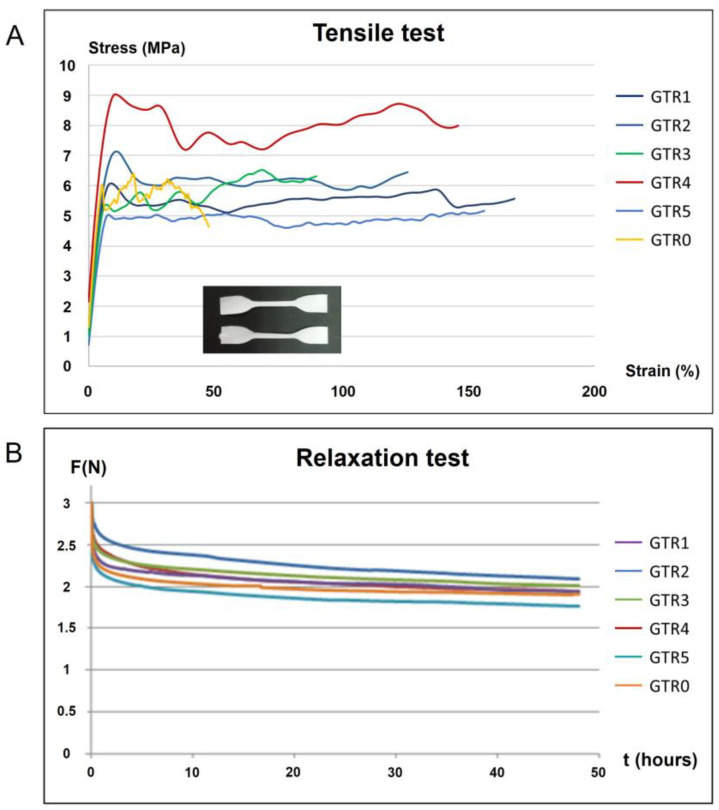
(**A**) Representative stress–strain curves under tensile loading and an image of the dumbbell shaped specimens used for tensile testing (inset). (**B**) Relaxation curves (force decay over time) for all membranes tested.

Time-independent and time-dependent mechanical properties of the GTR membranes were determined utilizing tensile and relaxation testing (Table 2). Figure 7A shows representative stress–strain curves where the membrane specimens are loaded up to fracture under tensile loading, and Figure 7B shows force–decay curves over time (relaxation).

Only the GTR5 membrane showed a significantly higher modulus of elasticity as compared to the control (GTR0), while the GTR1 and GRT2 membranes showed the highest ultimate tensile strength (UTS) in comparison to GTR0. All membranes showed a tendency to increase the capacity of plastic deformation, with the GTR2 and GTR4 membranes illustrating significant differences as compared to GTR0. In general, the results of tensile testing indicated that the deposition of further nanofibrous layers on the cast membrane tends to have a beneficial effect on the mechanical properties tested, with the most profound effect on plastic deformation capacity. The initial strength of the developed GTR membranes under investigation is comparable to that of commercially available resorbable membranes that are used for many years in the clinical practice, including membranes based on either synthetic polymers [65] or natural collagen [66,67]. Specifically, the UTS mean values of the fabricated GTR membranes ranged from 5.6 to 9.6 MPa, values similar to that of the poly-dl-lactic/co-glycolic acid-based membrane Resolut XT (11.7 MPa) [65].

As far as relaxation testing is concerned, no statistically significant differences were recorded among the developed GTR membranes. The percentile reduction of the initial force in 48 h reached approximately 35%, which indicates that sufficient available force remains even after 48 h. The absence of statistically significant differences indicates that the deposition of additional fibrous layers does not have any effect on the time-dependent property of relaxation.

### 2.6. Evaluation of the Growth and Attachment of PDL Cells Seeded on the GTR Membranes

**Figure 8:**
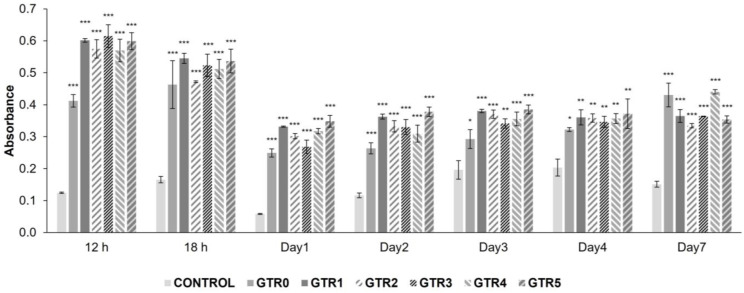
PDL cells’ growth on the developed GTR membranes and the control (CNT, plain culture plate). Bars represent means +/− standard deviation of the absorbance from MTT assay after 12 h, 18 h, 1 day, 2 days, 3 days, 4 days, and 7 days in culture. Data derived from three repetitions of the experiment, analyzed with one way ANOVA and subsequent Dunnett 2-sided *t*-tests, when appropriate. * *p* < 0.05; ** *p* < 0.005; *** *p* < 0.0001. Detailed statistics can be found in Table S1.

The biocompatibility of the fabricated membranes was evaluated in vitro using primary cultures of human PDL cells. The results of the MTT colorimetric assay through culture days 1 to 7 showed that all membranes (GTR1–5 and GTR0) support the growth and proliferation of PDL cells. As shown in Figure 8, the MTT absorbance was significantly increased during the 7 day period in all GTR membranes, as compared to the control group (PDL cells grown on plain culture plate), denoting increased proliferation and growth of the PDL cells seeded on the fabricated membranes (detailed statistical data are reported in Table S1).

**Figure 9:**
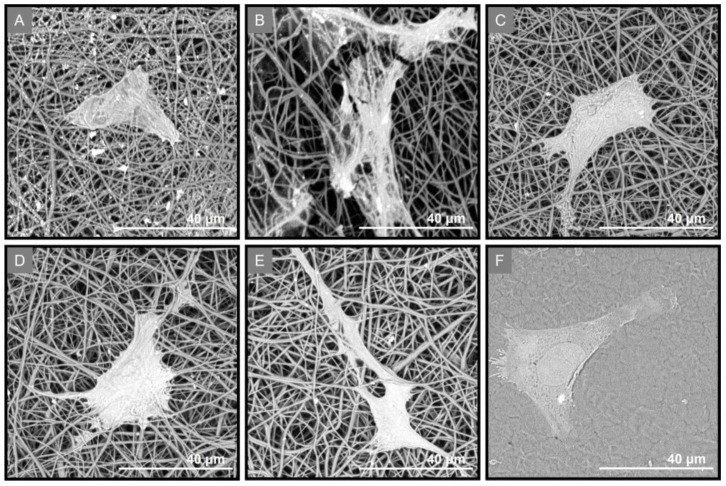
SEM images at three days post seeding of PDL cells on (**A**) GTR1, (**B**) GTR2, (**C**) GTR3, (**D**) GTR4, and (**E**) GTR5 membranes and (**F**) GTR0 (the cast membrane).

PDL cells’ attachment on the fabricated membranes was investigated using SEM analysis. Three days post seeding, the cells exhibited a flattened shape and adhered to all GTR membranes, including the cast membrane GTR0 (Figure 9).

### 2.7. Evaluation of the Effect of the GTR Membranes on the Osteo-Induction of PDL Cells

**Figure 10:**
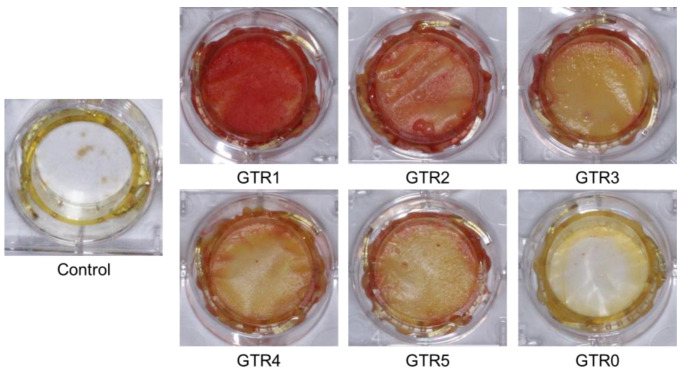
Representative culture plates of alizarin red-stained human PDL cells seeded on the GTR1–5 membranes, the cast membrane (GTR0), and the control (plain culture plate) after 1 week in osteo-inductive medium.

The results of the Ca^+2^ release study showed that the fabricated GTR membranes could act as a Ca^+2^ source for at least 3 weeks, facilitating osteoblasts’ growth and bone regeneration. We further examined the ability of these membranes to support and/or enhance the osteo-induction process of membrane-seeded PDL cells in the presence of a PDL osteo-inductive medium. Alizarin red staining of PDL cells after 1 week in osteogenic medium showed that osteo-induction was boosted earlier in cells seeded on the GTR1–5 membranes, as compared to cells seeded on GTR0 or on the control (plain culture plate) (Figure 10). The relative competence of the developed GTR membranes to support the osteo-induction process appeared to be GTR1 > GTR2 > GTR3 = GTR4 = GTR5. However, the sustained release of Ca^+2^ from these membranes did not allow for the precise quantification of the alizarin red staining results.

**Figure 11:**
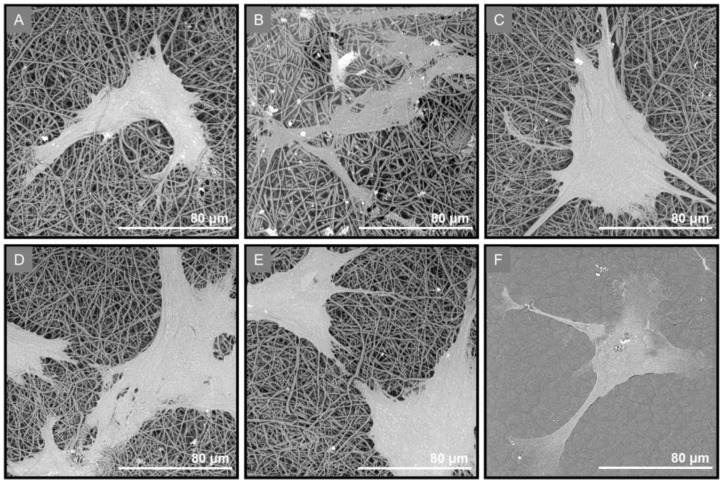
SEM images of PDL cells seeded on (**A**) GTR1, (**B**) GTR2, (**C**) GTR3, (**D**) GTR4, and (**E**) GTR5 membranes and (**F**) the cast membrane (GTR0) after 4 days culture in osteogenic medium.

The adherence of PDL cells on the GTR membranes during the osteo-induction was also evidenced by SEM imaging (Figure 11).

In order to investigate the molecular changes occurring in membrane-seeded PDL cells during the first week of osteo-induction, we measured the temporal expression levels of genes implicated in this process, at day 3 and day 7 post-induction. Specifically, we examined the expression of alkaline phosphatase (ALP) expressed in osteoprogenitor to immature osteoblasts, osteocalcin (OCN), a marker of mature osteoblasts [68], runt-related transcription factor 2 (RUNX2), upregulated throughout osteoblastic differentiation but downregulated in mature osteoblasts, and collagen type I alpha1 chain (COL1A) expressed in immature osteoblasts.

**Figure 12:**
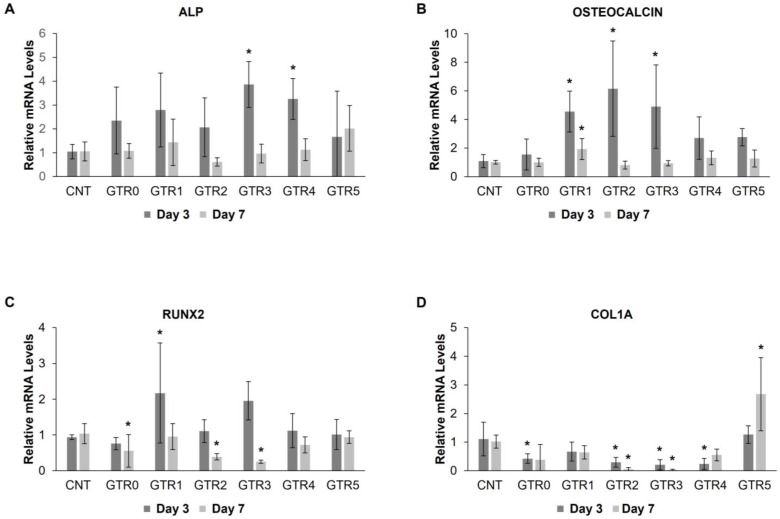
Expression of osteo-differentiation-related genes in GTR membranes-seeded PDL cells, after three and seven days of osteo-induction. The qRT-PCR data were analyzed using one way ANOVA and subsequent Dunnett 2-sided *t*-tests, when appropriate. * *p* < 0.05. Detailed statistics can be found in Table S2.

Statistical evaluation of qRT-PCR data showed a significant effect of the developed GTR membranes on the expression of the studied osteogenic markers, with the exception of ALP that was not significantly altered in PDL cells seeded on the various GTR membranes as compared to the control (Figure 12A) (detailed statistical data are reported in ). The expression of OCN, one of the latest markers of mature osteoblasts, was significantly increased at day 3 post-induction in PDL cells seeded on the GTR1, GTR2, and GTR3 membranes vs. cells in the control plate. This increase was retained in GTR1-cultured cells at day 7 post-induction (B). The pattern of OCN increased expression is in agreement with the potency for osteo-differentiation estimated via the alizarin red staining (GTR1 > GTR2 > GTR3). The expression of RUNX2 in GTR1-seeded cells was significantly higher vs. control at day 3 of osteo-induction, whereas its expression at day 7 was either similar to the control group (in the case of GTR1) or further reduced in cells seeded on the GTR2, GTR3, and GTR0 membranes (Figure 12C). RUNX2 is an early osteogenic marker and its reduced expression at day 7 of osteo-induction suggests an earlier process of osteo-differentiation in these cells. In the same line, the expression COL1A, an early marker of collagen matrix deposition prior to mineralization, was already reduced at day 3 post-induction in GTR2-, GTR3-, GTR4-, and GTR0-seeded cells. Of note, COL1A expression at day 7 was increased only in GTR5-seeded cells as compared to all other groups, implying a slower osteo-differentiation process (Figure 12D). Based both on the alizarin red staining and qPCR data, the GTR5 membrane appears to be the least efficient of the fabricated membranes in promoting osteo-differentiation of PDL cells in culture.

## 3. Materials and Methods

### 3.1. Materials

Polycaprolactone (PCL) (Mw 80,000), polyethylene oxide (PEO) (Mw 8,000,000 and Mw 900,000), dichloromethane (DCM), dimethylformamide (DMF), ethanol (EtOH), methanol (MeOH), and benzoylated dialysis tubing were purchased from Sigma-Aldrich (Darmstadt, Germany). Ca(OH)_2_ and NaOH were purchased from Lach-Ner (Neratovice, Czech Republic). All chemicals were of reagent grade and used directly without further purification.

### 3.2. Extraction and Characterization of Calcium Salt of Carrageenan (CG-Ca) and Sodium Salt of Carrageenan (CG-Na)

Dried biomass of the red alga *Chondrocanthus teedei*, harvested from in-land cultivation tanks in the island of Kefalonia, was kindly provided by the company Kefalonia Fisheries SA. The algal specimens were thoroughly cleaned of epiphytes and washed with tap water. Subsequently, they were air dried, ground to powder, and subjected to Soxhlet extraction using methanol to remove pigments.

For the extraction of CG-Ca, the depigmented, dried algal powder was soaked in distilled H_2_O (80 mL/g) and the pH of the solution was adjusted to 12–13 with the addition of a supersaturated aq. solution of Ca(OH)_2_. The alkaline solution was heated at 85–90 °C for 2 h under continuous stirring, and finally, the pH was adjusted to 8 with the addition of HCl. After filtration of the hot aqueous extract through a cotton cloth, the filtrate was evaporated under vacuum to half of its initial volume. Crude CG-Ca was precipitated with the addition of ethanol 96% (*v*/*v*) to the concentrated filtrate (twice the volume of the filtrate) and the resulting suspension was left overnight at 4 °C. The precipitate was separated by centrifugation (6000 rpm, 25 °C, 15 min) and successively washed with ethanol, sonicated in an ultrasonic bath, vacuum-filtered, and finally freeze dried overnight to afford crude CG-Ca as a greyish powder. Subsequently, CG-Ca was dissolved in distilled H_2_O, dialyzed against dd. H_2_O for 24 h using cellulose membranes and freeze dried overnight to obtain pure CG-Ca as yellowish spongy flakes. The isolated CG-Ca had a molecular weight distribution centered at 374 kDa and a sulfate content of 31.0% on a dry weight basis.

For the extraction of CG-Na, the depigmented, dried algal powder was soaked in NaOH (1 M, 150 mL/g) and heated at 85 °C for 3 h under continuous stirring. Subsequently, the pH was adjusted to 8 with the addition of HCl, and after filtration of the hot aqueous extract through cotton cloth, the same procedure as described for the isolation of CG-Ca was followed to afford pure CG-Na as yellowish spongy flakes. The isolated CG-Na had a molecular weight distribution centered at 278 kDa and a sulfate content of 28.4% on a dry weight basis.

Both CG-Ca and CG-Na isolated in the current study were identified as iota/kappa hybrids, characterized by a similar hybridization degree with 805/845 ratio values of 0.26 and 0.27, respectively, taking into account that the intensity ratio of 805 cm^−1^ (present in ι-carrageenans) over 845 cm^−1^ (present in both κ- and ι-carrageenans) absorption bands can be used as a parameter to determine the degree of iota/kappa hybridization [69,70,71].

### 3.3. Preparation of Calcium Poly(L-glutamate) (PG-Ca)

The synthesis of γ-benzyl-L-glutamate *N*-carboxyanhydride and poly(γ-benzyl-L-glutamate) was performed according to the literature [72]. Deprotection of the benzyl group was conducted with HBr/CH_3_COOH according to the literature [73]. Purification of the product by dialysis in dd. H_2_O provided the linear poly(L-glutamic acid). Dissolution of poly(L-glutamic acid) in dd. H_2_O and addition of the appropriate amount of Ca(OH)_2_ (1/2 equivalent of the PBLG-COOH groups) provided the final product PG-Ca.

### 3.4. Fabrication of the GTR Membranes

In total, five different GTR membranes were prepared and studied, among which two were tri-layer (GTR1, GTR2) and three were bi-layer (GTR3, GTR4, GTR5). In all cases, the outer layer of the GTR membranes consisted of a cast PCL film.

In the case of the GTR1 and GTR2 tri-layer membranes, the middle layer was composed of electrospun nanofibers of PG-Ca, while the inner layer comprised electrospun nanofibers based on CG-Ca or CG-Na, respectively. For the electrospinning of the PG-Ca, CG-Ca, and CG-Na, PEO was used as a polymeric carrier in order to enhance their electrospinninability into nonwoven fibrous membranes. For the preparation of the middle layer, PG-Ca was co-electrospun with PCL in order to ensure a stable cohesion with the cast outer layer and to enhance the stability of the nanofibers in an aqueous environment.

In the case of the GTR3, GTR4, and GTR5 bi-layer membranes, the inner layer consisted of electrospun nanofibers of CG-Ca/PG-Ca or CG-Ca or PG-Ca, respectively. For the electrospinning of the CG-Ca/PG-Ca, CG-Ca, and PG-Ca, PEO was used as a polymeric carrier in order to enhance their electrospinnability, while at the same time being co-electrospun with PCL in order to ensure a stable cohesion with the cast outer layer and to enhance the stability of the nanofibers in an aqueous environment.

#### 3.4.1. Preparation of the Cast Outer Layer

For the preparation of the outer layer, solvent casting was employed so as to create a cast layer on the surface of a rotating drum. To prepare the outer layer (cast membrane), PCL was dissolved at a concentration of 8% *w*/*v* in benzene at room temperature under stirring for 48 h to ensure the homogeneity of the solution. An appropriate volume of the PCL solution was transferred to a suitable container, and the drum was dipped in the polymer solution and allowed to rotate until the entire surface of the cylinder was covered by the polymer solution. Subsequently, the container containing the PCL solution was removed, and the drum was allowed to rotate under a stream of air until complete evaporation of the solvent and formation of a thin membrane on its surface. The outer layer served also as the control membrane (GTR0) in the analyses of the fabricated membranes.

#### 3.4.2. Preparation of the Spinning Solutions and Electrospinning

All spinning solutions were prepared under stirring for 24 h to ensure their homogeneity. The spinning solution of PCL was prepared at room temperature, whereas all the aqueous spinning solutions were prepared at 60 °C. For the preparation of the PCL spinning solution, PCL was dissolved at a concentration of 10% w/v in DCM/DMF 8:2 *v*/*v*. The CG-Ca/PEO and the CG-Na/PEO spinning solutions were prepared by dissolving CG-Ca or CG-Na, respectively, at a concentration of 1% *w*/*v* in distilled H_2_O followed by the addition of PEO (Mw 900,000) at a concentration of 4% *w*/*v*. The PG-Ca/PEO spinning solution was prepared by dissolving PG-Ca at a concentration of 4% *w*/*v* in distilled H_2_O followed by the addition of PEO (Mw 8,000,000) at a concentration of 4% *w*/*v*. The CG-Ca/PG-Ca/PEO spinning solution was prepared by dissolving CG-Ca at a concentration of 1% *w*/*v* in distilled H_2_O followed by the addition of PG-Ca at a concentration of 4% *w*/*v* and PEO (Mw 8,000,000) at a concentration of 4% *w*/*v*.

Electrospinning was conducted using a γ-High Voltage Research DC power supply generator of 50 kV maximum voltage (Gamma High Voltage Research, Ormond Beach, FL, USA). The spinning solutions were loaded into 10 mL disposable syringes fitted with stainless steel blunt needles (23G). The syringes were mounted on a horizontally positioned programmable syringe pump (Harvard PHD 2000, Harvard Apparatus, Holliston, MA, USA) and the produced nanofibers were deposited on a RC-6000 drum collector (NaBond Technologies, Hong Kong) rotating at 400 rpm. Temperature and relative humidity were 22 ± 2 °C and 65 ± 5%, respectively.

#### 3.4.3. Preparation of the Tri-Layer GTR1 Membrane

For the fabrication of the middle layer of the tri-layer membrane GTR1, the PCL, and PG-Ca/PEO spinning solutions were co-electrospun on the surface of the outer layer (PCL cast membrane) using an antiparallel setup with the syringes mounted on two horizontally opposed programmable syringe pumps to ensure the homogeneity of the blended polymer fibers. A small volume of the PCL spinning solution was initially electrospun alone to ensure cohesion between the deposited fibers and the cast membrane. The feeding rate and tip-to-collector distance of the PCL spinning solution were fixed at 2.5 mL/h and 16 cm, respectively, whereas the feeding rate and tip-to-collector distance of the PG-Ca/PEO spinning solution were adjusted at 5 mL/h and 27 cm, respectively. Electrospinning was conducted with the applied voltage fixed at 27 kV. For the fabrication of the inner layer of the tri-layer membrane GTR1, the CG-Ca/PEO spinning solution was electrospun on the surface of the middle layer with the applied voltage, feeding rate, and tip-to-collector distance fixed at 27 kV, 3 mL/h, and 30 cm, respectively.

#### 3.4.4. Preparation of the Tri-Layer GTR2 Membrane

For the fabrication of the middle layer of the tri-layer membrane GTR2, the PCL, and PG-Ca/PEO spinning solutions were co-electrospun on the surface of the outer layer (PCL cast membrane) using an antiparallel setup with the syringes mounted on two horizontally opposed programmable syringe pumps to ensure the homogeneity of the blended polymer fibers. A small volume of the PCL spinning solution was initially electrospun alone to ensure the cohesion between the deposited fibers and the cast membrane. The feeding rate and tip-to-collector distance of the PCL spinning solution were fixed at 2.5 mL/h and 16 cm, respectively, whereas the feeding rate and tip-to-collector distance of the PG-Ca/PEO spinning solution were adjusted at 5 mL/h and 27 cm, respectively. Electrospinning was conducted with the applied voltage fixed at 27 kV. For the fabrication of the inner layer of the tri-layer membrane GTR2, the CG-Na/PEO spinning solution was electrospun on the surface of the middle layer with the applied voltage, feeding rate, and tip-to-collector distance fixed at 27 kV, 3 mL/h, and 30 cm, respectively.

#### 3.4.5. Preparation of the Bi-Layer GTR3 Membrane

For the fabrication of the inner layer of the bi-layer membrane GTR3, the PCL and CG-Ca/PG-Ca/PEO spinning solutions were co-electrospun on the surface of the outer layer (PCL cast membrane) using an antiparallel setup with the syringes mounted on two horizontally opposed programmable syringe pumps to ensure the homogeneity of the blended polymer fibers. A small volume of the PCL spinning solution was initially electrospun alone to ensure the cohesion between the deposited fibers and the cast membrane. The feeding rate and tip-to-collector distance of the PCL spinning solution were fixed at 2.5 mL/h and 16 cm, respectively, whereas the feeding rate and tip-to-collector distance of the CG-Ca/PG-Ca/PEO spinning solution were adjusted at 5 mL/h and 27 cm, respectively. Electrospinning was conducted with the applied voltage fixed at 27 kV.

#### 3.4.6. Preparation of the Bi-Layer GTR4 Membrane

For the fabrication of the inner layer of the bi-layer membrane GTR4, the PCL and CG-Ca/PEO spinning solutions were co-electrospun on the surface of the outer layer (PCL cast membrane) using an antiparallel setup with the syringes mounted on two horizontally opposed programmable syringe pumps to ensure the homogeneity of the blended polymer fibers. A small volume of the PCL spinning solution was initially electrospun alone to ensure the cohesion between the deposited fibers and the cast membrane. The feeding rate and tip-to-collector distance of the PCL spinning solution were fixed at 1.5 mL/h and 16 cm, respectively, whereas the feeding rate and tip-to-collector distance of the CG-Ca/PEO spinning solution were adjusted at 3 mL/h and 26 cm, respectively. Electrospinning was conducted with the applied voltage fixed at 27 kV.

#### 3.4.7. Preparation of the Bi-Layer GTR5 Membrane

For the fabrication of the inner layer of the bi-layer membrane GTR5, the PCL and PG-Ca/PEO spinning solutions were co-electrospun on the surface of the outer layer (PCL cast membrane) using an antiparallel setup with the syringes mounted on two horizontally opposed programmable syringe pumps to ensure the homogeneity of the blended polymer fibers. A small volume of the PCL spinning solution was initially electrospun alone to ensure the cohesion between the deposited fibers and the cast membrane. The feeding rate and tip-to-collector distance of the PCL spinning solution were fixed at 2.5 mL/h and 16 cm, respectively, whereas the feeding rate and tip-to-collector distance of the PG-Ca/PEO spinning solution were adjusted at 5 mL/h and 27 cm, respectively. Electrospinning was conducted with the applied voltage fixed at 27 kV.

### 3.5. Scanning Electron Microscopy (SEM)

For the morphological characterization of the GTR membranes, the samples were precoated with a conductive layer of sputtered gold (samples with cells were examined without sputter coating) and examined using a PhenomWorld (Thermo Fischer Scientific, Waltham, MA, USA) desktop scanning electron microscope with a tungsten filament (10 kV) and a charge-reduction sample holder. The average fiber diameter of the nanofibers was determined by evaluating at least 100 measurements per sample using the embedded image analysis software (Phenom Pro Suite v2.3/Fibermetric).

Additionally, the PDL cells’ adherence on the fabricated membranes following seeding was detected with SEM. A total of 7,000 cells were cultured on the GTR membranes for 3 or 4 days. The cell-seeded membrane samples were fixed for 1 h using 2% (*v*/*v*) glutaraldehyde in 0.1 M cacodylate buffer (pH 7.4). The samples were afterwards transferred to 0.1 M cacodylate buffer containing 7% sucrose for 10 min, dehydrated with 50%, 70%, 80%, 95%, and 100% (*v*/*v*) ethanol solutions (two times, for 10 min each time, at 4 °C) and air dried before SEM analysis.

### 3.6. Fourier Transform Infrared Spectroscopy (FTIR)

FTIR spectra were recorded using the attenuated total reflection (ATR) method on a Bruker Alpha II (Billerica, MA, USA) FTIR spectrometer.

### 3.7. Thermogravimetric Analysis (TGA)

Thermogravimetric analyses (TGA) of the samples were performed using a TGA 55 Thermogravimetric Analyzer (TA Instruments, New Castle, DE, USA) from 27 to 600 °C at a heating rate of 10 °C/min under a 25 mL/min nitrogen flow.

### 3.8. Degradation Rate Study

To determine the degradation rate of the prepared membranes, the membranes were cut into pieces and weighed to record their initial weight (W_0_). After sterilization under UV, they were placed into vials and immersed in a simulated saliva solution (10 mg/mL) of pH 7.2 [74] and kept in an incubator at 37 °C. At different time intervals (1, 7, 14, 21, and 28 days), the membranes were removed and dried in a vacuum oven for 24 h at room temperature and weighed to record their final weight (W_t_). Four replicates for each membrane at each time point were measured.

The weight loss (%) of the membranes at each time point was calculated according to the equation:

$$Membrane\;Weight\;Loss(\%)=\frac{W_{0}-W_{t}}{W_{0}}\times 100$$

To calculate the weight loss of the fibrous layers for each membrane at each time point, the remaining fibrous layers—after the measurement of the membranes’ final weight (W_t_)—were removed by thorough rinsing with dd. H_2_O, and the cast film was dried in a vacuum oven for 24 h at room temperature and weighed to record the weight of the cast film (W_CF_).

The weight loss (%) of the fibrous layers of the membranes at each time point was calculated according to the equation:

$$Fibers\;Weight\;Loss(\%)=\frac{W_{0}-W_{t}}{W_{0}-W_{CF}}\times 100$$

### 3.9. Ca^+2^ Release Study

For the calcium release study, the membranes were cut into pieces and weighed. After sterilization under UV, they were placed into vials and immersed in a simulated saliva solution (10 mg/mL) of pH 7.2 [74] and kept in an incubator at 37 ℃. At different time intervals (1, 2, 7, and 21 days), the membranes were removed, and the concentration of free Ca^+2^ in the saliva solution was measured using the calcium assay kit ab272527 (Abcam, Cambridge, UK) according to the manufacturer’s instructions. Four replicates for each membrane were analyzed. The absorbance of the samples at 612 nm was recorded using an Infinite M200 PRO TECAN plate reader (Männedorf, Zürich, Switzerland). Calibration curves for Ca^+2^ were prepared using solutions with concentrations ranging from 0 to 200 μg/mL of Ca^+2^ that were prepared using a calcium standard solution diluted in appropriate volumes of dd. H_2_O.

### 3.10. Mechanical Properties of the GTR Membranes

Time-independent and time-dependent mechanical properties of the fabricated membranes were determined utilizing tensile and relaxation testing. Seven dumbbell shaped specimens were cut off for each GTR membrane and placed in a universal tensile testing machine (Tensometer Aegis 10, Monsanto, UK). The crosshead speed was set at 25 mm/min and stress–strain diagrams were recorded. Then, modulus of elasticity (E), ultimate tensile strength (UTS), and plastic strain were determined from each curve. As far as the determination of force reduction over time (relaxation) is concerned, seven rectangular shaped specimens (3 mm × 50 mm) were prepared for each membrane and then placed in a low-force-load cell machine connected to a PC measuring force every 30 s. The initially applied force was set at 3N and its reduction over time was recorded for 48 h, since initial studies indicated that the plateau phase was reached much sooner.

The collected data were tested for the presence of outliers employing the 1.5xIQR (InterQuartile Rule), normality (Shapiro–Wilk), and homoscedasticity (Brown–Forsyth). For normally distributed data, comparisons were carried out with one-way ANOVA, whereas for data failing to pass normality tests, the nonparametric one-way ANOVA on Ranks (Kruskal–Wallis) test was used. In all cases, Tukey post hoc multiple comparison tests were used to allocate differences among groups. The level of statistical significance for all tests was set at a = 0.05. Statistical analysis was carried out employing SigmaPlot v 14 software (Systat Software Inc., San Jose, CA, USA).

### 3.11. Biocompatibility of the GTR Membranes

#### 3.11.1. Cell Culture

The biocompatibility of the GTR membranes was tested in primary cultures of human periodontal ligament (hPDL) cells isolated from healthy permanent premolars that were extracted for orthodontic reasons in the Department of Oral and Maxillofacial Surgery, School of Dentistry, National and Kapodistrian University of Athens. The teeth were collected, upon written consent, from six donors 10–22 years old, and the study protocol was approved by the Ethics Committee of the School of Dentistry, National and Kapodistrian University of Athens. PDL tissue was washed in phosphate buffer saline (PBS) supplemented with antibiotics and antifungals, centrifuged, and resuspended in Dulbecco’s Modified Eagle’s Medium (DMEM). The tissue was digested with collagenase type I (Stem Cells Technologies, Vancouver, Canada) for 1 h at 37 °C and then cultured in DMEM supplemented with 10% fetal bovine serum (FBS), 100 units/mL penicillin/streptomycin and 0.25 μg/mL amphotericin (Thermo Fisher Scientific, Waltham, MA, USA) at 37 °C and 5% CO_2_. At passage 4, an equal number of cells from each donor were aliquoted and stored at −80 °C. In all experimental procedures, a mixture of PDL cells from all donors was used by co-culturing cells from the frozen PDL aliquots. PDL cells at passage 5–6 were used for all analyses.

#### 3.11.2. Treatment of the GTR Membranes

Pieces of the GTR membranes (40 × 40 mm and 15 × 15 mm for 6-well and 24-well plates, respectively) were sterilized under UV before use. The membrane pieces were stabilized at the bottom of the culture wells with sterile crown inserts (Sigma Aldrich, Darmstadt, Germany).

#### 3.11.3. Cytocompatibility of the GTR Membranes and Proliferation of PDL Cells

Cytocompatibility and proliferation of PDL cells seeded on the fabricated GTR membranes were evaluated using the MTT colorimetric assay during the first 7 days in culture. Briefly, PDL cells were plated on the fabricated GTR membranes in 24-well plates (3000 cells/well) in DMEM supplemented with 10% FBS, 100 units/mL penicillin/streptomycin and 0.25 μg/mL amphotericin. After 12, 18, 24, 48, 72, 96, and 168 h in culture, DMEM was removed and MTT reagent (1 mL/well, final concentration 0.5 mg/mL, Thermo Fisher Scientific, Waltham, MA, USA) was added in the wells for 4 h. The supernatants were discarded, and formazan crystals were diluted with DMSO. Absorbance was measured at 570 nm with a VersaMax ELISA Microplate Reader (Molecular Devices LLC, San Jose, CA, USA). The absorbance of the samples at 690 nm was used as a reference. The results were compared with the control group (PDL cells grown on plain culture plate) and evaluated using one way ANOVA and subsequent Dunnett 2-sided *t*-tests, when appropriate. Increased MTT absorbance indicated membranes’ biocompatibility and subsequent cell growth.

### 3.12. Osteo-Differentiation of PDL Cells Seeded on the GTR Membranes

For osteo-differentiation experiments, PDL cells of passage 4 were seeded on the fabricated GTR membranes in 6-well plates (5000 cells/well). After one day in culture, the medium was supplemented with 50 μg/mL ascorbic acid (Sigma Aldrich, Darmstadt, Germany), 10^−8^ M dexamethasone (Sigma Aldrich, Darmstadt, Germany), and 10 mM sodium β-glycerophosphate (Calbiochem-Novagen, Darmstadt, Germany). The osteo-inductive medium was replaced every 2 days. After 7 days, the supernatants were removed and the cells on the membranes were washed twice with 1X PBS (Gibco, Waltham, MA, USA). The cells were fixed with neutral formalin 10% for 30 min at room temperature and washed with dd. H_2_O. Alizarin red S solution (2%) was added to stain the cells for 20 min at room temperature, and the membrane-seeded cells were washed 3 times with dd. H_2_O.

### 3.13. qRT-PCR Analysis

NucleoZOL and NucleoSpin RNA Set for NucleoZOL (Macherey-Nagel, Düren, Germany) were used to extract total RNA from PDL cells seeded on membranes GTR 1–5 and the cast membrane GTR0 and from PDL cells grown on plain culture plate (control) at 3 and 7 days after incubation with osteo-inductive medium. Subsequent reverse transcription into cDNA was performed using PrimeScript RT Reagent Kit (Perfect Real Time, TAKARA, Shiga, Japan). Quantitative real-time RCR was performed using iTaq Univer SYBR Green Supermix (BIO-RAD, Dubai, United Arab Emirates) to quantify expression levels of mRNA for alkaline phosphatase (ALP), osteocalcin (OCN), runt-related transcription factor 2 (RUNX2), and collagen type I alpha1 chain (COL1A1). GAPDH was used as the normalization housekeeping gene. The primers used can be found in Table S3. Specificity of PCR products was evaluated by melting curve analysis. Fold changes in gene expression were calculated using the 2^−ΔΔCT^ method [75]. Statistics for each time point (day 3 and day 7 post-induction) were performed with one way ANOVA (SPSS 22.0). Subsequent post hoc comparisons of all other groups with the control group were carried out using Dunnett two-sided *t*-test. Differences between groups were considered statistically significant for *p* values < 0.05.

## 4. Conclusions

The treatment of periodontitis still remains a challenge, and the development of effective GTR/GBR membranes is attracting significant attention as a potential solution. Capitalizing on the well-known potential of carrageenans for bone regeneration by promoting the attachment and proliferation of osteoblasts, bi- and tri-layer nanofibrous GTR membranes based on carrageenans and other biocompatible polymers were designed, fabricated, and characterized. All GTR membranes showed adequate stability for up to 28 days of incubation at 37 °C in a simulated saliva solution, while exhibiting a sustained release of Ca^+2^ for at least three weeks, demonstrating their potential to act as a continuous source of calcium ions for promoting the growth of osteoblasts and bone regeneration. The initial strength of the developed GTR membranes was adequate and comparable to commercially available resorbable ones that are currently used in clinical practice. Our findings on the gene expression of relevant osteo-differentiation markers during osteogenic induction of PDL cells supported the potential of the designed GTR membranes, especially the tri-layer one composed of calcium salt of carrageenan and calcium poly(L-glutamate), to promote osteo-induction at an earlier time point.

## Figures and Tables

**Table 1:** Polymeric composition of the developed bi- and tri-layer GTR membranes.

Membrane	Outer Cast Layer	Middle Nanofibrous Layer	Inner Nanofibrous Layer
GTR1	PCL	PG-Ca/PEO ^1^, PCL	CG-Ca/PEO ^2^
GTR2	PCL	PG-Ca/PEO ^1^, PCL	CG-Na/PEO ^2^
GTR3	PCL	-	CG-Ca/PG-Ca/PEO ^1^, PCL
GTR4	PCL	-	CG-Ca/PEO ^2^, PCL
GTR5	PCL	-	PG-Ca/PEO ^1^, PCL
GTR0	PCL	-	-

^1^ PEO: Mw 8,000,000; ^2^ PEO: Mw 900,000.

**Table 2:** Mechanical properties of the GTR membranes tested with mean values and standard deviation in parenthesis, and with median and 25% and 75% percentiles in brackets in the case of an absence of normality, where E is modulus of elasticity, UTS is ultimate tensile strength, strain refers to plastic deformation, and RAS refers to percentile reduction of initial force. The presence of different superscripts indicates statistically significant differences among groups (*p* < 0.05).

GTR Membranes	E (MPa)	UTS (MPa)	Strain (%)	RAS (%)
GTR1	117.6 (13.6) ^ABC^	7.6 (0.4) ^C^	78 [72, 120] ^ABC^	32.2 (2.2) ^A^
GTR2	118.4 (15.7) ^AB^	9.6 (1.6) ^A^	138 [136, 320] ^A^	34.1 (5.7) ^A^
GTR3	90.5 (8.2) ^AB^	5.6 (0.4) ^Β^	100 [89, 118] ^ABC^	37.0 (3.6) ^A^
GTR4	88.0 (20.9) ^C^	5.9 (1.2) ^ΒC^	150 [96, 159] ^AB^	33.4 (4.8) ^A^
GTR5	128.3 (26.8) ^B^	6.5 (1.1) ^ΒC^	70 [59, 86] ^BC^	37.7 (2.3) ^A^
GTR0	97.3 (12.3) ^AC^	5.7 (1.1) ^B^	45 [36, 53] ^C^	33.3 (6.3) ^A^

## Data Availability

The data that support the findings of this study are available from the corresponding authors upon request.

## Supplementary Materials

The following supporting information can be downloaded at: https://www.mdpi.com/article/10.3390/md21110565/s1, Figure S1: degradation profiles of membranes and their fibrous layers; Table S1: statistical data for proliferation and growth of PDL cells; Table S2: statistical data for qRT-PCR results; Table S3: primers used for qRT-PCR analysis.
