# Supplementary material for: Development of Bi- and Tri-Layer Nanofibrous Membranes Based on the Sulfated Polysaccharide Carrageenan for Periodontal Tissue Regeneration
Source: Mar Drugs. 2023 Oct 28;21(11):565. doi: 10.3390/md21110565 (PMC10671875; doi:10.3390/md21110565)
Supplement: Supplementary file 1 [file marinedrugs-21-00565-s001.zip › marinedrugs-2677831-supplementary.pdf]

Supplementary Materials for

Development of Bi- and Tri-Layer Nanofibrous Membranes based on the Sulfated Polysaccharide Carrageenan for Periodontal Tissue Regeneration

Stefanos Kikionis [1,†], Konstantina Iliou [1,†], Aikaterini G. Karra [2], Georgios Polychronis [3], Ioannis Choinopoulos [4], Hermis Iatrou [4], George Eliades [3], Efthymia Kitraki [2], Ioulia Tseti [5], Spiros Zinelis [3], Efstathia Ioannou [1] and Vassilios Roussis [1,*]

[1] Section of Pharmacognosy and Chemistry of Natural Products, Department of Pharmacy, National and Kapodistrian University of Athens, Panepistimiopolis Zografou, 15771 Athens, Greece; skikionis@pharm.uoa.gr (S.K.); ilioukonstantina@outlook.com (K.I.); eioannou@pharm.uoa.gr (E.I.)

[2] Department of Basic Sciences, School of Dentistry, National and Kapodistrian University of Athens, 11527 Athens, Greece; aikaterini.g.karra@gmail.com (A.K.); ekitraki@dent.uoa.gr (E.K.)

[3] Department of Biomaterials, School of Dentistry, National and Kapodistrian University of Athens, 11527 Athens, Greece; gpolisg@yahoo.gr (G.P.); geliad@dent.uoa.gr (G.E.); szinelis@dent.uoa.gr (S.Z.)

[4] Industrial Chemistry Laboratory, Department of Chemistry, National and Kapodistrian University of Athens, Panepistimiopolis Zografou, 15771 Athens, Greece; ichoinop@chem.uoa.gr (I.C.); iatrou@chem.uoa.gr (H.I.)

[5] Uni-Pharma S.A., 35 Kalyftaki Str., 14564 Kifissia, Greece; jtsetis@uni-pharma.gr (I.T.)

† These authors contributed equally to this work.

* Correspondence: roussis@pharm.uoa.gr (V.R.)

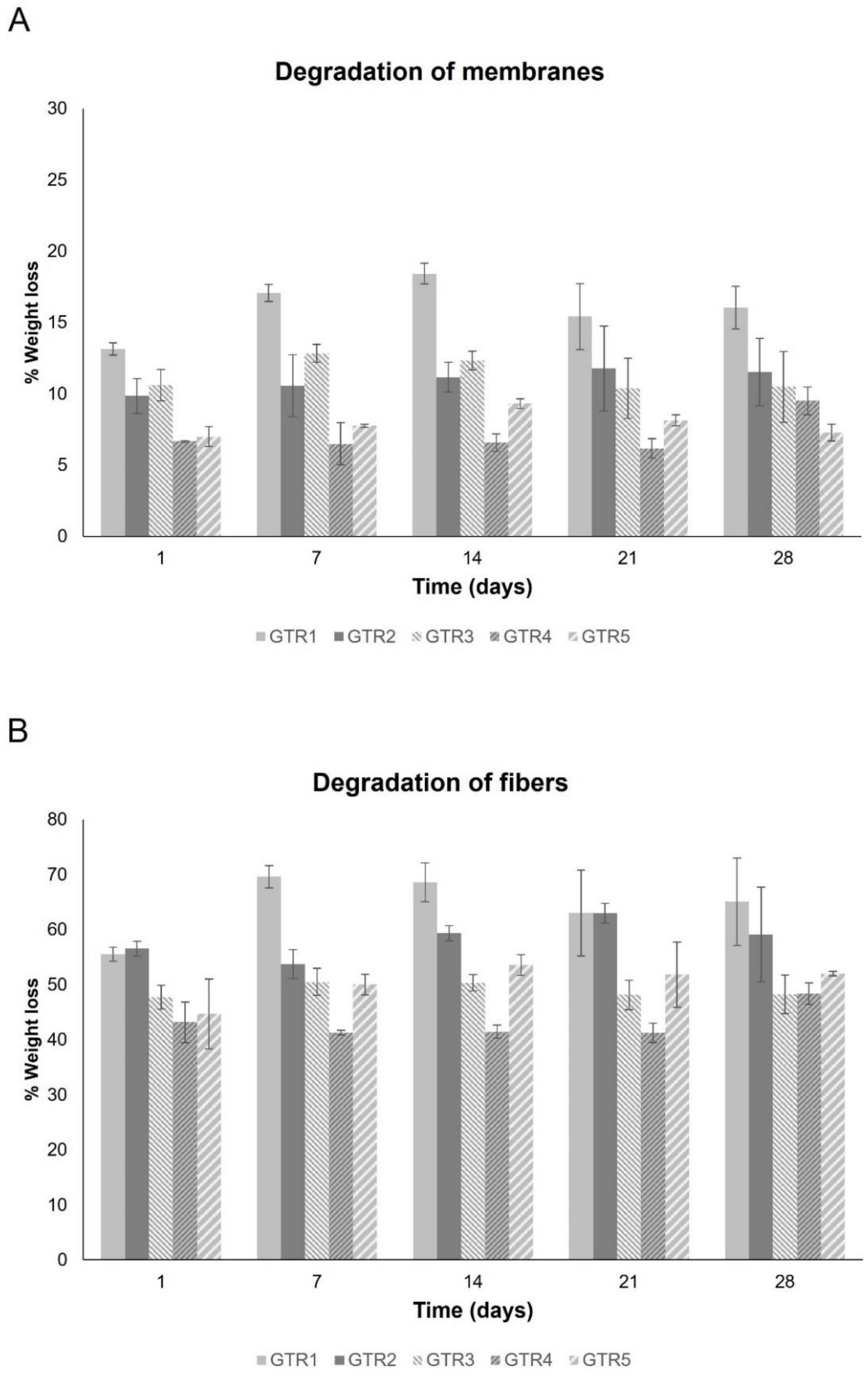

Figure S1. Degradation profiles of (**A**) GTR1, GTR2, GTR3, GTR4 and GTR5 membranes and (**B**) their fibrous layers.

Table S1. Statistical data for MTT results relating to the proliferation and growth of PDL cells. F and P values are provided for the one way ANOVA. Subsequent post hoc P values of the Dunnett t 2-sided test, refer to comparisons of GTR membrane groups with the Control group, for each time point. Statistical significance is accepted for $p < 0.05$

time point	F value	*p* value	post hoc *p* values *vs.* CONTROL					
			GTR0	GTR1	GTR2	GRT3	GTR4	GTR5
12 H	$F_{(6,20)}$= 50.41	0.000	0.000	0.000	0.000	0.000	0.000	0.000
18H	$F_{(6,20)}$= 13.25	0.000	0.000	0.000	0.000	0.000	0.000	0.000
DAY 1	$F_{(6,20)}$= 63.70	0.000	0.000	0.000	0.000	0.000	0.000	0.000
DAY 2	$F_{(6,20)}$= 24.88	0.000	0.000	0.000	0.000	0.000	0.000	0.000
DAY 3	$F_{(6,20)}$= 11.66	0.000	0.000	0.000	0.001	0.000	0.000	0.018
DAY 4	$F_{(6,20)}$= 5.81	0.003	0.002	0.002	0.005	0.003	0.001	0.018
DAY 7	$F_{(6,20)}$= 30.77	0.000	0.000	0.000	0.000	0.000	0.000	0.000

Table S2. Statistical data for qRT-PCR results. F and P values are provided for the one way ANOVA. Subsequent post hoc P values of the Dunnett t 2-sided test, refer to comparisons of GTR membrane groups with the control group, for each gene and time point. Statistical significance is accepted for $p < 0.05$.

Gene - day	F value	*p* value	post hoc *p* values *vs.* CONTROL					
			GTR0	GTR1	GTR2	GTR3	GTR4	GRT5
ALP-DAY 3	$F_{(5,35)}$= 1.902	0.125	--	--	--	--	--	--
ALP-DAY 7	$F_{(5,38)}$= 3.536	**0.012**	1.000	0.789	0.670	1.000	1.000	0.051
OCN-DAY 3	$F_{(5,40)}$= 4.776	**0.002**	0.997	**0.021**	**0.000**	**0.014**	0.529	0.541
OCN-DAY 7	$F_{(5,41)}$= 5.174	**0.001**	1.000	**0.004**	0.913	1.000	0.659	0.808
RUNX2-DAY 3	$F_{(5,37)}$= 4,14	**0.005**	0.995	**0.013**	0.995	0.07	0.995	1.0
RUNX2-DAY 7	$F_{(5,39)}$= 5.677	**0.001**	**0.025**	0.991	**0.002**	**0.001**	0.225	0.979
COL1A1-DAY 3	$F_{(5,39)}$= 7.571	**0.000**	**0.003**	0.087	**0.000**	**0.000**	**0.000**	0.935
COL1A1-DAY 7	$F_{(5,39)}$= 18.139	**0.000**	0.231	0.716	**0.025**	**0.046**	0.522	**0.000**

Table S3. Primers used for qRT-PCR analysis.

Gene	forward primers	reverse primers
gapdh	5′-TCTTCACCACCATGGAGAA-3′	5′-ACTGTGGTCATGAGCCCTT-3′
ALP	5′-GACCTCCTCGGAAGACACTC-3′	5′-TGAAGGGCTTCTTGTCTGTG-3′
OCN	5′-CGCAGCCACCGAGACACCAT-3′	5′-AGGGCAAGGGGAAGAGGAAAGAA-3′
RUNX2	5′-CCGCACGACAACCGCACCAT-3′	5′-CGCTCCGGCCCACAAATCTC-3′
COL1A1	5′-TGCTCGTGGAAATGATGGTG-3′	5′-CCTCGCTTTCCTTCCTCTCC-3′
